# Extramedullary Hematopoiesis in the Cerebral Dura Resulting in Fatal Brain Herniation in a Patient With Secondary Myelofibrosis: A Case Report

**DOI:** 10.1155/crh/5796734

**Published:** 2026-07-14

**Authors:** Takayuki Fujio, Yuichi Hasegawa, Yasumitsu Kurokawa, Akinori Sugaya, Shingo Ishiguro, Shoichi Mitsuhashi, Hiroshi Kojima, Hitoaki Saitoh, Mitsuo Hori

**Affiliations:** ^1^ Department of Hematology, Ibaraki Prefectural Central Hospital, Kasama, Japan, pref.ibaraki.jp; ^2^ Ibaraki Clinical Education and Training Center, University of Tsukuba Hospital, Kasama, Japan, tsukuba.ac.jp; ^3^ Department of Medical Oncology, Ibaraki Prefectural Central Hospital, Kasama, Japan, pref.ibaraki.jp; ^4^ Department of Diagnostic Pathology, Ibaraki Prefectural Central Hospital, Kasama, Japan, pref.ibaraki.jp

## Abstract

Extramedullary hematopoiesis (EMH) is a compensatory process that supplements defective hematopoiesis and is frequently observed in myeloproliferative neoplasms (MPNs). Liver, spleen, and lymph nodes are the most common sites of EMH, whereas its occurrence in the intracranial space is extremely rare. We experienced a case of EMH which developed in cranial dura mater after irradiation against massive splenomegaly. As general condition of the patient did not allow us pathological examination, the diagnosis was confirmed by postmortem autopsy.

## 1. Introduction

Extramedullary hematopoiesis (EMH) is characterized by blood cell production occurring outside the bone marrow. It is usually a compensatory response in hematologic disorders. The most common underlying diseases are chronic anemias such as thalassemia and myeloproliferative neoplasms (MPNs) including primary myelofibrosis (PMF) and polycythemia vera (PV) [1]. Liver, spleen, and lymph nodes are common sites of EMH, whereas its occurrence in the intracranial space is extremely rare. Due to the invasiveness of diagnostic procedures for intracranialEMH, pathological proof is seldom available. Here, we report a case of secondary myelofibrosis (SMF) with extensive EMH in the cranial dura mater that led to fatal brain herniation.

## 2. Case Presentation

A 57‐year‐old female was referred to our hematology department because of mild leukocytosis and thrombocytosis in March 2007. Physical examination revealed splenomegaly palpable 8 cm below the left costal margin. Based on bone marrow morphological findings and the absence of the major BCR‐ABL transcript, she was suspected as having MPN. Because she had been suffering from manifested symptoms of schizophrenia and refused further examination, bone marrow biopsy was not performed. Her leukocytosis and thrombocytosis were thereafter followed at a nearby psychiatric hospital with no treatment. She also had a history of right middle cerebral artery infarction at the age of 56 years, which resulted in left hemiparesis. In April 2011 at 71 years of age, she was referred again because of worsening leukocytosis. Laboratory findings were as follows: white blood cell (WBC) count 43,900/μL, hemoglobin (Hb) level 12.2 g/dL, platelet (Plt) count 214 × 10^3^/μL, LDH 1131 U/L, and ferritin 12.7 ng/mL. PCR analysis of peripheral blood cells revealed *JAK2* V617F mutation. Although erythrocytosis was not prominent, likely due to concomitant iron deficiency, she was diagnosed as having PV. As her psychological condition did not allow us to perform bone marrow biopsy throughout the clinical course, the disease could not be differentiated from PMF. Treatment with hydroxyurea was initiated to control leukocytosis. The spleen index evaluated by CT scan in May 2012 was 383 cm^2^ (normal, less than 40 cm^2^). Thereafter, her splenomegaly progressed gradually, which reached to infraumbilical level in 2015. To relieve abdominal discomfort, low‐dose splenic irradiation (6 gray in 6 fractions and 2 fractions/week) was performed in May 2015, June 2018, and April 2020. As bone marrow failure had progressed by August 2018, she became red blood cell transfusion‐dependent. Although bone marrow biopsy was not performed, we assumed that myelofibrosis was the cause of the bone marrow failure.

In June 2021, she was admitted because of somnolence and gait disturbance. Her consciousness was mildly blurred (E4V4M6 by Glasgow Coma Scale). Laboratory data were as follows: WBC 55,500/μL with 4% blasts and 12% immature myeloid cells, Hb 9.4 g/dL, Plt 102 × 10^3^/μL, LDH 895 U/L, creatinine 0.92 mg/dL, and CRP 3.11 mg/dL. Brain CT revealed extensive subdural high‐density tumor‐like lesions around the falx and bilateral parietal areas (Figure 1). Biopsy of the intracranial tumor‐like lesion was planned. However, her consciousness level and respiratory condition deteriorated, and she died of brain herniation on the 21st day of hospitalization.

**FIGURE 1:**
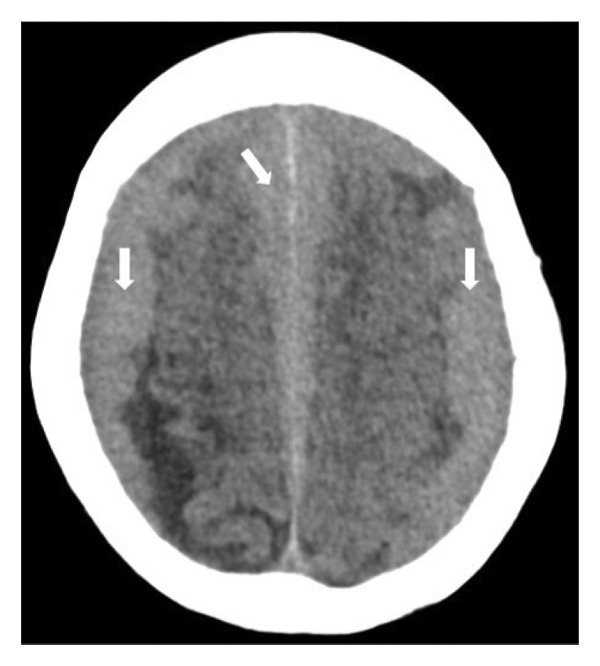
Noncontrast CT showing extensive subdural lesions around the falx cerebri and bilateral parietal areas (white arrows).

Autopsy was performed, which revealed extensive EMH in the thickened cranial dura mater (Figure 2), as well as in the spleen, liver, lung, gallbladder, stomach, small intestine, and right ovarian duct. Bone marrow fibrosis was prominent. These findings suggest that brain herniation attributable to intracranial EMH, which advanced in conjunction with the progression of myelofibrosis, was the cause of death in this patient.

**FIGURE 2:**
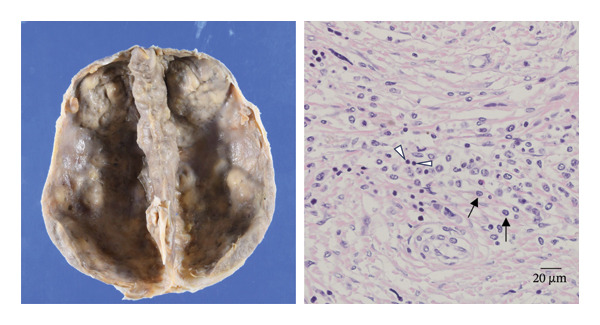
Autopsy findings of the cranial dura mater. Macro (left panel) and micro (right panel) observations. Right panel shows proliferation of hematopoietic cells in the cranial dura mater (hematoxylin‐eosin staining, x400). Black arrows and white arrow heads show myeloid and erythroid cells, respectively.

## 3. Discussion

The currently hypothesized mechanism underlying EMH is as follows. First, normal myeloid tissue extends into adjacent tissues, thereby initiating hematopoiesis, as observed in hemoglobinopathies. Second, when bone marrow function is impaired by hematological disorders, such as MPN and myelofibrosis, circulating stem cells seed extramedullary tissues and establish hematopoiesis [2]. Although the liver, spleen, and lymph nodes are common sites of EMH, it also occurs in other organs, including the lung, kidney, adrenal gland, thymus, and gastrointestinal tract [3–5]. Rare cases of EMH in intracranial locations, including the cranial meninges, spinal meninges, paraspinal tissues, and intracerebral regions, have been reported [6–8]. Although intracranial EMH is often asymptomatic, it can become symptomatic because of mass effect. When intracranial pressure increases, as observed in intracranial tumors, various symptoms, such as hemiplegia, impaired consciousness, headache, and diplopia, may develop [9–12]. In the present patient, an expanding intracranial EMH lesion led to a fatal increase in intracranial pressure. To our knowledge, this is the first report of death due to brain herniation which is attributable to intracranial EMH.

Massive splenomegaly is not uncommonly observed in patients with myelofibrosis. A previous report showed that splenomegaly exceeding 10 cm below the costal margin is observed in 30.7% of overt PMF, 35.1% of post‐PV SMF, and 14.7% of postessential thrombocythemia SMF [13]. Although JAK inhibitors are the first‐choice treatment for patients with symptomatic massive splenomegaly, splenectomy and splenic irradiation still remain as a second‐choice treatment for patients who are not eligible for JAK inhibitors [14]. However, previous reports have documented the occurrence of EMH in atypical sites, such as the skin, mediastinum, and intracranial space, in splenectomized patients with myelofibrosis or thalassemia [12, 15]. In these cases, absence of the spleen, the primary site of EMH, may have accelerated compensatory hematopoiesis in extramedullary tissues. Similarly, we consider that splenic irradiation in the present patient hampered pluripotent activity of stem cells in the spleen, which might have accelerated EMH in other sites such as the dura mater, liver, lung, gallbladder, stomach, small and large intestines, and ovarian duct. Indeed, autopsy findings revealed that EMH in the spleen is minimal in comparison with that observed in other sites.

Diagnosis of intracranial EMH is a clinical challenge. Craniotomy biopsy is highly invasive, and conventional image findings obtained by CT and MRI are not specific for EMH. Although we planned craniotomy biopsy in the present patient, deterioration of the patient’s general condition did not allow us to make antemortem diagnosis. As a noninvasive diagnostic approach for EMH, bone marrow scintigraphy may be useful [16]. Indeed, a previous case report indicated that bone marrow scintigraphy with ^111^In chloride, in combination with histopathological examination of other biopsy‐accessible EMH sites, was useful for the diagnosis of intracranial EMH [17]. The patient was successfully treated by combination of ruxolitinib administration and 15 Gy radiation to the intracranial mass.

It is important for hematologists to recognize that patients with myelofibrosis may develop intracranial EMH, which may result in serious central nervous system dysfunction. Although definitive diagnosis requires histopathological examination, the procedure poses a substantial clinical challenge.

## 4. Conclusion

We report a patient with SMF who developed extensive EMH in the cranial dura mater, leading to fatal brain herniation. Intracranial EMH is a rare complication of MPN, but it should be considered in patients with MPNs who present with neuropsychiatric symptoms. Although splenectomy or splenic irradiation is a choice of treatment for massive splenomegaly in patients with myelofibrosis, it may provoke EMH outside of the spleen. We thus believe that treatment options which impair splenic hematopoietic activity should be considered carefully in patients with myelofibrosis associated with massive splenomegaly.

## Funding

This study was not supported by any sponsor or funder.

## Consent

Written informed consent for both the autopsy and the publication was obtained from the patient’s legal guardian.

## Conflicts of Interest

The authors declare no conflicts of interest.

## Data Availability

Data sharing is not applicable to this article as no datasets were generated or analyzed during the current study.
